# A Case of Gastrostomy

**Published:** 1889-08

**Authors:** Jas. McFadden Gaston

**Affiliations:** Professor of the Principles and Practice of Surgery, Southern Medical College, Atlanta, Ga.


### A CASE OF GASTROSTOMY.

By J. McFADDEN GASTON, M. D.,

Professor of the Principles and Practice of Surgery, Southern Medical College, Atlanta, Ga.

The patient was a white man, thirty-five years old, who had suffered for several years with great difficulty in swallowing, and ultimately was unable to swallow even water, so that he was nourished by enemata, while under the treatment of Dr. A. C. Moreland. Finally, on the 8th of July, the patient was turned over by him to Dr. J. McFadden Gaston, who with the assistance of Drs. W. D. and B. W. Bizzell, Crichton, C. D. Roy and Sims, performed the operation of gastrostomy on July 13th. Dr. Moreland was not present on account of his sickness. An oblique incision of four inches, one inch below the cartilages of the ribs on the left side, was carried through all the parietal structures, and after the slight flow of blood had ceased, the peritoneum was opened. The fingers of the left hand being introduced, the stomach was brought into the external wound, and two strong silk ligatures passed at a distance of an inch and a half through the front wall of the greater curvature, which were held by an assistant, while an incision of an inch was made through all the coats of the stomach. The forefinger of the left hand being passed through this up to the cardiac orifice, a bougie of a half an inch diameter was, with moderate pressure, carried through the constriction, about two inches above this orifice.

An effort to carry a tube from the mouth some weeks previous to the operation, had not succeeded in passing the stricture, but the bougie passed from below.

This being effected, the incision in the coats of the stomach was brought in contact with the inner and upper portion of the external opening and after closing the peritoneum of the outer and lower portion with the continuous cat-gut suture, the edges in contact with the gastric peritoneum were attached in like manner to it, at a distance of half an inch from the margin of the incision in the coats of the stomach. The tegumentary and muscular structures of the lower and outer portion of the parietes were now united by deep interrupted suture of iron-dyed silk. The margins and opposite edges of incision of the stomach were brought together by alternate stitches of silk and cat-gut without tying, until after the insertion of a silver canula with flanges inside and outside, around which the tissues were closed by knotting the thread. The tube having a diameter of half an inch, in which a rubber stopper was inserted, terminated within by a disc of an inch in diameter and without by one, of an inch and a half in diameter, so as to be self-retaining, if the parts should heal around it.

As the patient was able to swallow fluids from the dilatation by the bougie, small quantities of milk with lime water and milk punch were allowed for three days, but there was regurgitation or belching of most all that he took. On the evening of the 16th, peptonized milk and lime water was thrown into the stomach through the canula, but it was returned by contraction of the stomach. He continued to have nourishment thrown up into the colon every four hours with addition of Tincture of Chinchona.

The urine had to be drawn off by catheter, but collected about the quantity *excreted* before operating. The temperature had been subnormal prior to the operation and did not exceed 100° Far. for the first two days afterwards, but on the 17th it had reached 103¼ and at 2 o'clock p. m. on that day he succumbed.

An examination four hours after death found the external wound agglutinated and without suppuration at any point, but there was a slight aperture on tearing open the external parts found in the peritoneal line near the junction with the gastric walls on the outer or lower side, which indicated a lack of union by adhesive inflammation. The attachment of the margins of the stomach to the edges of the parietes was complete, and after the stitches were cut away could only be broken up by the use of considerable force.

The constriction in the œsophagus extended for two inches, with thickening and induration of all its coats, but admitted readily in the rather irregular canal the handle of a scalpel, proving thus that there was no mechanical obstacle to the passage of fluids after the bougie had been introduced on the occasion of the operation. Below the thickened stricture, the tissues of the canal were attenuated and apparently tending towards desintegration. The specimen has been left with Dr. B. W. Bizzell for examination, under the supposition that scirrhus degeneration will be discovered by the microscopic investigation.

The statistics of this class of cases are so very unfavorable as to offer little encouragement for the success of gastrostomy, and it is desirable that the operation shall be undertaken before the vital forces are materially impaired.

